# Identification of Corrosive Volatile Compounds Found in the Headspace of Chicken Noodle Soup Retorted in Metal Cans

**DOI:** 10.1155/2023/9662709

**Published:** 2023-08-17

**Authors:** Yajun Wu, Ken Ruffley, Elliot Dhuey, Christopher M. Hadad, Melvin A. Pascall

**Affiliations:** ^1^Department of Food Science and Technology, The Ohio State University, Columbus, OH 43210, USA; ^2^PPG Industries Inc., 500 Techne Center Dr. Milford, OH 45150, USA; ^3^Department of Chemistry and Biochemistry, The Ohio State University, Columbus, OH 43210, USA

## Abstract

This study investigated the development of volatile compounds in the headspace of canned chicken noodle soup (and sought to develop appropriate testing methods). The primary objective of this study was to identify compounds in the soup that were responsible for the initiation of the corrosion in the cans. The long-term goal of these studies is to develop an efficient method to investigate how headspace volatile compounds in foods could cause corrosion defects in metal cans and how these could be corrected without undermining the quality and safety of the food. To determine and to evaluate the volatile compounds in the canned soups, selected ion flow tube–mass spectrometry (SIFT-MS) was used. The coatings of the tested cans were carefully stripped off and analyzed using this SIFT-MS method. High levels of sulfur-containing volatile compounds and organic acids were detected in both the soups and the coatings. It was concluded that during the retorting of the sealed cans filled with chicken soup, sulfur-containing volatile compounds formed and entered the headspace of the tested cans and interacted with the coating, leading to the formation of blackened stains.

## 1. Introduction

On a global scale, commercially available canned foods have become an important part of our daily diet. Most food cans are made from tin-plated steel, although some foods are packaged in tin-free steel cans. On the contrary, most beverage cans are made from aluminum. However, corrosion in metal cans may occur if the material is not adequately protected from aggressive foods, especially if conditions inside the cans are conducive for such reactions. Metal corrosion is defined as an electrochemical reaction in which the metal surface is oxidized [1]. Corrosion in canned foods starts when aggressive chemical compounds migrate into the interior walls of the cans and react with the base metal [2]. To minimize corrosion in food cans, most containers are coated with polymeric compounds, such as epoxy resins, lacquers, enamel, and polyesters. Corrosion in canned foods causes major failures including coating delamination, reaction of the food electrolytes with the base metal, excessive migration of metal compounds to the food products, and hydrogen swelling of the cans [3]. The migration of metal compounds from the corroded can towards the packaged food could become a safety issue if the metal concentration reaches a critical limit. Exposure to high concentrations of tin (>250 mg kg^−1^) can irritate the gastrointestinal tract and cause short-term symptoms including nausea, vomiting, and diarrhea [4]. The literature reports similar findings for aluminum migration from cans to packaged foods [5]. Additionally, metallic migration brings an end to shelf life when it causes an alteration in the taste and color of the canned food. Therefore, polymeric coatings are commonly applied to the internal metal surface of certain food cans in order to delay the onset of corrosion.

One common coating type used on metal packaging is epoxy resins. Because of the highly cross-linked nature of epoxy compounds, they form tough heat-resistant coatings that are excellent barriers to the formation of corrosion in metal packaging. Epoxy resins also show excellent adhesion to the base metal [6]. Among all types of epoxy resins, the most popular coating resins are synthesized through the condensation polymerization reaction between epichlorohydrin and bisphenol A (BPA) [7, 8]. Prior to 2010, 95% of food contact coatings in metal cans were epoxy types that contained BPA [9]. While the use of BPA-based epoxy coatings may provide excellent corrosion protection, the retorting conditions and the aggressive nature of certain foods are capable of accelerating the hydrolysis of the ester bonds in BPA-based polymers. This has the potential to cause small amounts of BPA to leach from the coating towards the packaged food [10]. With increasing public health concerns and governmental restrictions that limit the use of BPA, the food packaging industry is pushed to consider the use of BPA-free materials. In the United States, the most restrictive legislation that addresses BPA exposure (3 *μ*g/day) is in Proposition 65 in Title 27, California Code of Regulations, section 25805(b) [11].

This action against the use of BPA in food contact polymeric materials created the need to develop BPA-free compounds with corrosion-resistant properties. To accomplish this, it is essential to gain a detailed understanding of how the heat from retorting conditions can initiate changes to the chemical compounds in the processed foods, especially if these compounds are capable of initiating corrosion. According to the level of aggressiveness, canned foods are usually divided into four categories: (1) nonaggressive; (2) medium aggressive; (3) highly aggressive; (4) sulfur-rich products [2]. Nonaggressive products are dried foods which contain little moisture. These include foods such as dried fruit, pasta, and powdered products. Medium aggressive products are foods that contain organic acids, which contribute to medium or acidic pH. Highly aggressive products (pH 3 to 4.6) also contain organic acids when compared with medium aggressive products (pH 4.7 to 5). Sulfur-rich products are foods that have a high level of sulfur, often in the protein source. Fish and meat are typical sulfur-rich products, because the protein component is the major source of sulfur [12].

**Figure 1:**
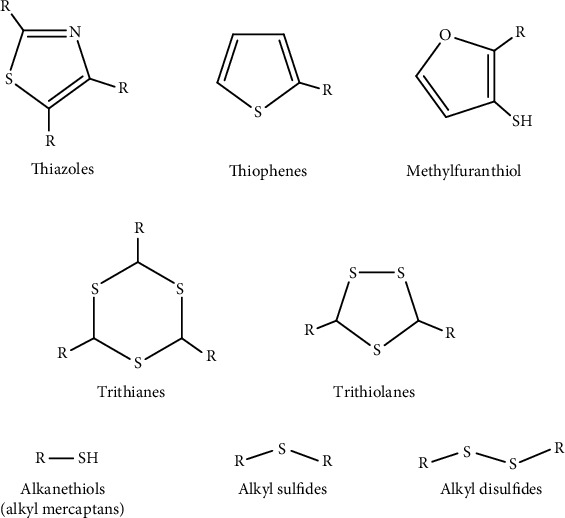
Representative sulfur-containing compounds in meat [13].

Cysteine is a sulfur-containing amino acid found in the flesh of certain animals, including chicken. In fact, the meat flavor is closely associated with sulfur-containing heterocyclic compounds. During heat treatment, cysteine interacts with inosine-5′-monophosphate (IMP) and produces 2-methyl-3-furanthiol, which is identified as the most important chemical compound responsible for the meaty flavor of chicken broth. Various sulfur derivative compounds that are released due to cysteine degradation are shown in Figure 1 [13]. These sulfur-containing compounds were reported to cause lacquer failure in food metal containers. Kontominas et al. [14] conducted research on common defects in the interior walls of metal cans containing tuna in soybean oil and found that lacquer adhesion failure caused by sulfides from fish allowed acidic compounds in the containers to migrate towards the internal metal surface, and this initiated discoloration and corrosion. Black deposits are sometimes observed on the internal walls of defective cans that contain sulfur-rich products [3, 15]. These black deposits are defined as “sulfur staining,” which develops when FeS clusters form inside the defective cans [16]. This study investigated the types of volatile compounds produced in chicken noodle soups that were retorted in metal cans coated with an epoxy corrosion-resistant liner. Qualitative and quantitative analyses were performed in order to understand how these volatile compounds initiated corrosion in the samples that were tested in this study.

This study began by using a selected ion flow tube–mass spectrometry (SIFT-MS) method to investigate the headspace volatile compounds released from the soups that were tested. As a real-time measurement technique, SIFT-MS monitors the concentrations of volatile compounds in humid air [17]. It is widely applied in several areas of research, including breath analysis, environmental research, and volatiles released by food products [18, 19]. Volatile organic compounds that are associated with the corrosion process can be identified using SIFT-MS.

## 2. Materials and Methods

### 2.1. Materials and Ingredients

Three types of packaging were tested in this study: metal cans, glass jars, and retort pouches. The metal cans were 284 ml (10 oz), 211 × 400 D&I (draw and wall ironed) in size, and supplied by PPG Industries Inc. (Mason, Ohio). The glass jars were ball 227 ml (8 oz) regular mouth mason jars, purchased from a local grocery store in the Columbus, Ohio, area. The retort pouches were 227 ml (8 oz), 201 × 264 mm in dimension, and 178 *μ*m thick and made from metallized polyethylene terephthalate (PET). They were supplied by VacUpack Canada Marketing Inc. (Renton, Washington). The chicken broth, canned chicken breast, raw chicken breast, egg noodle, soup base, salt, frozen carrots, fresh celery, dried parsley, and dried onion were purchased from local grocery stores in the Columbus, Ohio, area. The chicken fat was obtained from grocery stores in Cleveland, Ohio. The modified starch was purchased from WinCrest Bulk Foods (Moundsville, New York). The potassium chloride (P9541-500 g) was purchased from Sigma-Aldrich, Co. (St. Louis, Missouri). Cysteine chloride obtained from Sigma-Aldrich, (St. Louis, MO) was used as a standard in this study. To prepare the buffer solution, potassium biphosphate (KH_2_PO_4_) and sodium biphosphate (Na_2_HPO_4_) were both purchased from Fisher Scientific (Waltham, MA).

### 2.2. Experimental Design

All soup samples were made in the food processing pilot plant in the Department of Food Science and Technology at The Ohio State University. This study was designed to examine the volatile organic compounds that were produced by heating the chicken noodle soup by (1) identifying the headspace volatile compounds in glass jars filled with chicken noodles (control) and in coated metal cans; (2) determination of the origin of the identified volatile compounds by systematic elimination of the chicken noodle soup ingredients; and (3) understanding how cysteine and other acids-initiated corrosion in the sample cans.

The recipe was adjusted to create samples with at least one or more missing ingredients. The individual ingredients left out from each adjusted recipe at any one time were chicken broth, chicken breast, noodle/egg, soup base, potassium chloride (KCl), salt (NaCl), modified food starch, frozen carrots, fresh celery, dried parsley, dried onion, and chicken fat. Thus, the main ingredients in these cans were chicken noodle soup, chicken breast, frozen carrot, and fresh celery. The control was one set of soup with all of the ingredients, but heat treated in glass jars. The chicken noodle soup group contained all of the ingredients. The chicken breast group included precooked chicken breast, chicken broth, potassium chloride (KCl), and sodium chloride (NaCl). The carrot group contained frozen carrot, chicken broth, KCl, and NaCl. The composition of the celery group was fresh celery, chicken broth, KCl, and NaCl. The broth and salt groups consisted of chicken broth, KCl, and NaCl.

Cysteine was used as an ingredient to replace chicken breast in this study. The cysteine treatment group contained cysteine, chicken broth, KCl, and NaCl. The cysteine buffer group contained cysteine and a buffer. All groups described above were also prepared and packaged in glass containers and tested by SIFT-MS analysis on day 0. A second control group was also prepared. This control had the same products but packaged in retort polymeric pouches. This was done to prevent possible corrosion in the cap of the glass jars from interfering with the accuracy of the results. Additionally, samples were prepared in which distilled water was used to replace the chicken broth in samples that contained chicken breast, frozen carrot, fresh celery, and noodles.

### 2.3. Soup Sample Preparation

The soup formulation is summarized in Table 1. The fresh celery and frozen carrots were hand chopped into pieces measuring 8 × 8 mm in size. The chicken breast, egg noodle, frozen carrot, and fresh celery were weighed and added to the packaging directly. Then, the liquid portion was added to a predetermined headspace (7.14 mm). The soup base, KCl, chicken fat, dried parsley, and dried onion were mixed with the chicken broth and heated to 76.7°C for 5 minutes. The modified starch was weighed and well mixed with about 50 mL chicken broth before adding it to the broth mixture.

To prepare the cysteine-buffer solution, 0.5 g of cysteine chloride (Sigma-Aldrich, St. Louis, MO) was added to 1 L of buffer solution. The buffer solution consisted of 3.56 g KH_2_PO_4_ (Fisher Scientific, Waltham, MA) and 1.41 g Na_2_HPO_4_ (Fisher Scientific, Waltham, MA) in 1 L of distilled water. The final pH of the cysteine-buffer solution was adjusted to 7.0. All soup samples were hot filled into the packaging. The glass jars were tightened by hand to a torque of 9 Nm. This was to ensure that no leakage in the glass jars took place during the study. The metal cans were sealed using a can seamer (Dixie Canner Co., Athens, GA). A seam scope was used to evaluate the double seam for these cans which required a 1.90–2.16 mm body hook, 1.78–2.29 mm cover hook, and >1.14 mm overlap. Adjustments were made to the can seamer in order to obtain proper double seams. The average double seam measurements of the tested cans used in this study were body hook =1.90 mm; cover hook =2.03 mm; overlap =1.30 mm, and these were within the required range for properly sealed cans. After sealing, all samples were shaken to ensure proper mixing of the ingredients.

**Figure 2:**
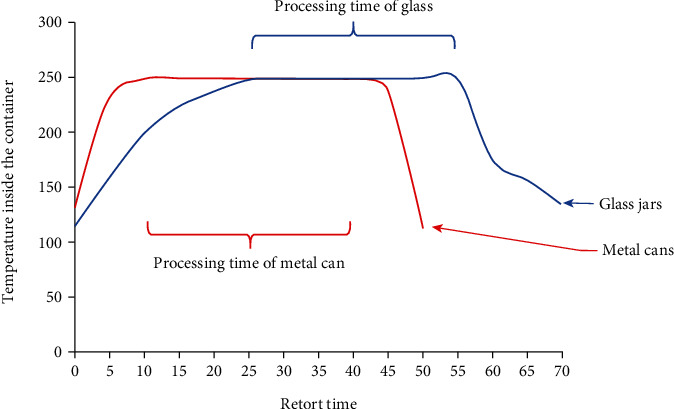
Heat penetration studies for glass containers (blue) and metal cans (red).

The canned soup samples were loaded into baskets immediately after seaming and then conveyed into a still vertical retort (Dixie, Athens, GA). After closing the retort, the samples were heated to a hold temperature of 121°C. The glass jars, plastic pouches, and metal cans required different processing times because the heat transfer rates of these materials were different. Therefore, the samples packaged in plastic, glass, and cans were processed separately. To ensure that all samples received an adequate processing time, heat penetration studies were conducted, and examples for glass and metal containers are shown in Figure 2. These studies were done by attaching thermocouples to specially built cans and glass jar covers designed for heat penetration analysis. The temperature/time profiles of the samples were obtained by plotting the temperature and processing times required to heat the “cold spot” of the containers to 121°C. The cold spot is the center of the container or the last part of the container to reach the required processing temperature. The processing time for the metal cans was determined to be 30 minutes. The processing time for the glass containers was determined to be 40 minutes.

After processing and cooling, all canned soup samples were stored at 40°C prior to testing for corrosion because higher storage temperatures are known to promote the corrosion reaction at an accelerated rate [20]. After the containers were opened, the soups were tested for volatile compounds using the SIFT-MS method. Additionally, the internal coatings of the cans were subsequently stripped off and analyzed by the SIFT-MS method for organic volatiles that migrated to it from the soup. The coatings were removed using a proprietary electrolysis method. This was done by immersing an empty test can (with the coating) in a 10% solution of sodium bicarbonate in distilled water in a glass tank. This was connected to the positive pole of a 12-volt battery. The negative pole of the battery was connected to an uncoated steel metallic reference measuring 4 cm wide and 6 cm long. After approximately 2 hours, the polymeric coating delaminated from the metallic wall of the can. The coating was then rinsed in deionized distilled water, dried, and stored for further analysis as a stand-alone film.

### 2.4. SIFT-MS Analysis

A Voice200® instrument (Syft Technologies Ltd., Christchurch, New Zealand) was used to analyze the volatiles released into the headspace of the test containers. Prior to the SIFT-MS analysis, all samples were stored at 40°C after retorting for the time periods previously discussed. The control, chicken noodle soup, and cysteine buffer groups were opened and tested on days 20, 25, 30, and 35 after retorting. The broth and salt and celery groups were opened and tested on days 5, 10, 15, 20, and 25. The chicken breast, carrot, and cysteine groups were tested on days 5, 8, 11, and 14. The distilled water samples were tested right after retorting. These storage times were selected after preliminary results showed the average times when corrosion was likely to be visual in the different ingredient groups.

For the quantification of the volatile compounds in the soups, the method used was one published by [21]. The process began by adding 25.0 g aliquots of each soup in separate 500 mL Pyrex media storage bottles. Each bottle was capped with an open-top screw cap fitted with an airtight silicone septum and incubated in an 80°C water bath for one hour. Before analysis, all media glass bottles and septa were incubated at 100°C for 12 hours to remove any trace residual volatile compounds. The headspace volatile compounds in the glass bottles were quantified directly by coupling the bottles to the inlet port of the instrument using an 18-gauge, 3.8-cm-long stainless-steel piercing needle. Prior to scanning a sample, 50°C water was scanned to clean the loading tube of the SIFT-MS instrument. A blank analysis was done before and after each test, and the results were subtracted from the actual test results. Air was scanned between consecutive sample tests in order to clean the loading tube. All results were collected using the Syft VOICE-200 software. For the quantification of the volatile compounds in the can coating, the process began by adding 25.0 g aliquots of the coating from each soup can in separate 500 mL Pyrex media storage bottles. These were then tested similar to the quantification method reported for the soup above.

This analysis focused on the levels of two categories of compounds that were suspected of breaching the coating of the tested cans and initiating the corrosion reaction. These were (1) sulfur-containing volatile compounds and (2) organic acids.  Table 2 summarizes the information used to identify the volatile compounds of interest in the headspace of the bottles. The data collected represented the relative percentages of the volatile compounds of interest. Each soup sample was tested in triplicate. The intensities of the compounds in each sample were calculated from the average of six determinations (2 batches × 3 determinations/batch). The compounds in the soup and in the coating were identified based on the library that formed part of the Syft VOICE-200 software. These were also compared with the references reported in Table 2.

## 3. Results and Discussion

### 3.1. Identification of Headspace Volatile Compounds in Chicken Noodle Soup in Glass Jars and Metal Cans

Table 3 shows the results from the SIFT-MS analysis for the control group. This represented the chicken noodle soup packaged in glass jars. The soup samples were analyzed on days 0, 20, 25, 30, and 35. High levels of sulfur-containing volatile compounds were identified in these soup samples, including hydrogen sulfide, dimethyl sulfide, methyl mercaptan, propyl mercaptan, methional, and dimethyl trisulfide. [13] reported that these chemicals are typical breakdown products of sulfur-containing compounds such as cysteine and amino acids found in proteins. This occurred during the retorting and storage of the soup. High levels of organic acids were also found in the soup samples, including acetic, propionic, butyric, and isovaleric acids. These acids may have come from the carrots and celery. Vegetables, such as carrots and celery, are good sources of organic acids, including malic and citric acid. The Center for Food Safety and Applied Nutrition at FDA (2015) reported that canned carrots have a pH of 5.18-5.22, and cooked celery has a pH of 5.37-5.92. The highest percentages of sulfur-containing volatile compounds and organic acids were found in the soup samples that were opened on day 0 and their concentrations decreased during the storage period. These results suggested that these volatile compounds were primarily generated from the product during the retorting process. Dhuey et al. [26] and Leonard et al. [27] also reported the mechanism of food degradation during retorting.

Table 4 shows the SIFT-MS analysis results for the chicken noodle soup packaged in metal cans. The soup samples were analyzed on days 0, 20, 25, 30, and 35, while the coatings that were removed from the cans were analyzed on days 20, 25, 30, and 35. High levels of sulfur-containing volatile compounds and organic acids were identified in the soup samples. This result indicated that some of these volatile compounds were absorbed by the coating during the storage period. High intensities of sulfur-containing volatile compounds were found in the coating, including dimethyl sulfide, propyl mercaptan, methional, and dimethyl trisulfide. High intensities of acids, including acetic, propionic, butyric, and isovaleric acids, were also detected in the coating. It was apparent that these compounds penetrated the coating during retorting and storage (Figure 1). Corrosion was observed in the tested cans for this sample on day 25. The results also showed that the intensities of the acids decreased as the storage time increased. Thus, it appeared that these acids might have interacted with the metal underlining the coating. Previous studies indicated that high acidity from organic acids could accelerate corrosion in canned foods [28].

### 3.2. Origin of Volatile Compounds in Chicken Noodle Soup by Systematic Elimination of Ingredients

The SIFT-MS results for the chicken breast and the carrot groups are summarized in Tables 5 and 6, respectively. The soup samples were analyzed on days 0, 5, 8, 11, and 14, while the coatings were analyzed on days 5, 8, 11, and 14. Similar SIFT-MS results were obtained for these two groups. Both the chicken breast and the carrot group samples had the broth as the only common ingredient. High levels of sulfur-containing volatile compounds and organic acids were identified in the soup samples and the coatings. Corrosion was observed in the tested cans for both groups on day 11. Aggressive corrosion-initiating compounds can be released from certain foods when they are heat treated. These include organic acids which are known to accelerate corrosion reactions in metal cans [29]. From the results of our studies, it appears that sulfur-containing volatile compounds interacted with the coating of the tested cans. As a result, breaches (discontinuities) developed in the coating. This made it easier for the organic acids to migrate from the soup towards the metal surface. Canned sulfur-rich products found in canned fish, chicken, clams, and cheese are reported to be associated with corrosion which resulted after the coating adhesion failure [3].

Tables 7 and 8 show the SIFT-MS results for the celery and the broth groups, respectively. These soup samples were analyzed on days 0, 5, 10, 15, 20, and 25 of storage, while the coatings were analyzed on days 5, 10, 15, 20, and 15. Similar SIFT-MS results were obtained for these two groups. Both the celery and the broth groups had the broth ingredients as common additives. In Table 7, high levels of sulfur-containing volatile compounds and organic acids were also identified in these soup samples. For the coatings, the intensities of dimethyl trisulfide, acetic, and propionic acid volatile compounds were very low on day 5 but increased on day 10. Corrosion was observed in the tested cans for both groups on day 10. After this time, the intensities of these compounds decreased as storage time increased. These results suggested that sufficient levels of sulfur-containing volatile compounds were necessary to breach the coating of the tested cans and create channels for electrolytes and oxidants in the soup to interact with the base metal.

To isolate the ingredients that released the sulfur-containing volatile compounds and organic acids, the chicken broth was replaced by distilled water.  Table 9 shows the SIFT-MS results for the chicken breast, frozen carrot, fresh celery, and egg noodle groups. All soup samples were analyzed on day zero. The results show that chicken breast released the high levels of hydrogen sulfide, dimethyl sulfide, and methyl mercaptan sulfur-containing volatile compounds. It also appeared that frozen carrot released the highest levels of acetic acid, while for celery, it was propionic acid and 2-methylbutyric acid. Relatively high intensities of sulfur-containing volatile compounds and organic acids were also detected in the egg noodles. According to the ingredient label of the egg noodles, ferrous sulfate, thiamine mononitrate, and folic acid were additives in the product. These compounds appeared to have served as a good source of sulfur and acids during the retorting process.

### 3.3. Determination of the Roles of Cysteine and Acids in Initiating Corrosion

Table 10 summarizes the SIFT-MS results for the cysteine group. These soup samples were analyzed on days 0, 5, 8, 11, and 14, while the coatings were analyzed on days 5, 8, 11, and 14. High levels of organic acids and sulfur-containing volatile compounds were identified in the soup samples and in the coatings. The intensities of the sulfur-containing volatile compounds, especially hydrogen sulfide, in the cysteine group, were much higher when compared to the other groups, perhaps because the addition of cysteine provided a greater source for sulfur-containing volatile compounds formation. Cysteine gives rise to various sulfur-derived compounds during heat treatment. Hydrogen sulfide is one of those compounds. Black staining in the tested cans for the cysteine group was observed on day 8. This black staining could have developed when FeS forms inside the defective cans [14].

Table 11 summarizes the SIFT-MS results for the cysteine buffer group. These soup samples were analyzed on days 0, 20, 25, 30, and 35, while the coatings were analyzed on days 20, 25, 30, and 35. No obvious corrosion was found in the tested cans for the cysteine buffer group during the 35-day storage time. Sulfur-containing volatile compounds that had a high intensity included hydrogen sulfide and dimethyl sulfide. For the cysteine buffer group, cysteine was the only sulfur source in the package. Thus, less sulfur-containing volatile compounds formed during the heat treatment. The levels of organic acids in this group were much lower when compared to the other groups. The buffer solution in the package allowed the product to maintain a constant pH and prevented interaction between hydrogen ions and the base metal of the tested cans during the time of this study. Additionally, this test group had no broth, chicken, carrot, or celery. These results suggested that the pH of the product was important for the initiation of corrosion in the tested cans [28]. Testing with only the acids and salts would have been helpful in order to determine if these compounds by themselves would cause corrosion in the absence of the sulfur-containing compounds. This will be included in future studies that will continue the focus on corrosion in metal cans used to package tomato products.

Preliminary findings of this study surveyed various canned food items on the market and found that this type of corrosion was particularly problematic in cans filled with chicken noodle soup. It was postulated that the volatile compounds in the headspace of the cans could initiate the corrosion process. Corrosion localized to the headspace region of canned foods is one area of concern. Headspace is the unfilled space in the container between the top of the food and the underside of the lid. Headspace plays a major role in canning because it impacts the vacuum and overpressure of the can during retorting which in turn impacts the corrosion protection of the package [30]. As a result of these findings, it was necessary to develop a method to investigate the relationship between headspace volatile compounds and the corrosion defects in the canned chicken noodle soup.

The cysteine breakdown products may play an important role in creating breaches in the lacquer of the metal cans. These volatile compounds may have permeated the can coating before creating breaches, which allowed other aggressive compounds to attack the base metal. Thus, identifying volatile compounds released from the coatings at high concentration levels was crucial to our understanding of the corrosion initiation.

## 4. Conclusion

During the retorting of the sealed cans filled with chicken products, sulfur-containing volatile compounds formed and entered the headspace of the cans. These compounds had the potential to bond to the coating of the cans and create discontinuities. These discontinuities were studied, and the results were presented in another research paper using scanning electron microscopy-energy dispersive X-ray spectroscopy (SEM-EDS). However, this present study observed that no visual signs of corrosion occurred when cysteine with the buffer alone was tested. Thus, we concluded that a lower pH was essential for corrosion to occur.

This study demonstrated that SIFT-MS has the potential to effectively analyze headspace volatile compounds in metal cans. Due to limitations in the numbers of cans from the same batch that were provided by the partnering industrial company, testing of the cans with only salt and organic acids was not possible. This should be done in future work on this or similar projects. Also, a control set of samples should be tested in glass containers stored in an upside-down orientation. This will allow for the testing of volatiles that had not contacted the metal in the cap. Since the lid of the glass jar was made with metal and had a polymeric lining, it was unclear if this influenced the results. Future work should also include the following: (1) a sample group made with pure salt and pure acids; (2) analyze other types of canned food and identify the common compounds of interest that would initiate corrosion; and (3) analyze the headspace of the tested cans and identify the volatile compounds of interest without opening the cans.

## Figures and Tables

**Table 1:** Chicken noodle soup formulations.

Ingredients	Chicken noodle soup	
	% by weight	Weight per can (g)
Chicken broth	82.37	257.2
Soup base	0.85	2.7
Soup base sodium free	2.15	6.7
Chicken fat	0.05	0.2
Dried onion	0.07	0.2
Dried parsley	0.02	0.1
KCl	0.67	2.1
Modified starch	0.5	1.6
Chicken	3.75	11.7
Carrots	1.41	4.4
Celery	1.41	4.4
Noodles	6.75	21.1
Total	100	312.4

**Table 2:** Major volatiles, reagent ions, and masses of the compounds used in this study.

Compounds	Formula	Mass	Reagent	*m*/*z*	Reference
Sulfur					
Hydrogen sulfide	H_2_S	34	H_3_O^+^	35	a and e
Dimethyl sulfide	(CH_3_)_2_S	62	H_3_O^+^	63	a
Methyl mercaptan	CH_4_S	48	H_3_O^+^	49	a
Propyl mercaptan	C_3_H_8_S	76	No^+^	76	a
Methional	C_4_H_8_OS	104	No^+^	104	a, b, and d
Dimethyl trisulfide	C_2_H_6_S_3_	126	H_3_O^+^	127	a and d
2-Isobutylthiazole	C_7_H_11_NS	141	H_3_O^+^	142	c
Acid					
Acetic acid	C_2_H_4_O_2_	60	H_3_O^+^	61	a, b, and d
Propionic acid	C_3_H_6_O_2_	74	H_3_O^+^	75	a
Butyric acid	C_4_H_8_O_2_	88	H_3_O^+^	89	e
Isovaleric acid	C_5_H_10_O_2_	102	H_3_O^+^	103	a
2-Methylbutyric acid	C_5_H_10_O_2_	102	No^+^	103	c and d

^a^[22], ^b^[23], ^c^[24], ^d^[25], and ^e^Kumar and others, 2013.

**Table 3:** Relative percentages of volatile compounds of interest in the headspace of the chicken noodle soup sample packaged in glass jars (control).

Compounds	Day 0	Day 20	Day 25	Day 30	Day 35
Sulfur					
Hydrogen sulfide	0.6396 ± 0.1599	0.0362 ± 0.0031	0.0309 ± 0.0231	0.0471 ± 0.0139	0.0627 ± 0.0108
Dimethyl sulfide	95.9230 ± 7.9261	97.7833 ± 4.3666	97.4147 ± 7.8457	96.9527 ± 9.7869	97.1554 ± 9.4590
Methyl mercaptan	1.3854 ± 0.3864	0.0030 ± 0.0021	0.0380 ± 0.0139	0.0444 ± 0.0165	0.1048 ± 0.0212
Propyl mercaptan	0.4131 ± 0.1228	0.3257 ± .0905	0.3776 ± 0.0855	0.4247 ± 0.0826	0.3975 ± 0.0881
Methional	1.3431 ± 0.4445	1.4026 ± 0.2209	1.7730 ± 0.3649	2.1043 ± 0.5908	1.7852 ± .6061
Dimethyl trisulfide	0.2787 ± 0.0923	0.4269 ± 0.0449	0.3527 ± 0.0816	0.4010 ± 0.0760	0.4685 ± 0.0905
2-Isobutylthiazole	0.0171 ± 0.0106	0.0224 ± 0.0026	0.0131 ± 0.0062	0.0258 ± 0.0097	0.0258 ± 0.0085
Acid					
Acetic acid	2.50879 ± 0.6760	3.68494 ± 0.7097	2.99828 ± 0.5800	5.38308 ± 1.0911	12.01428 ± 5.1678
Propionic acid	13.70257 ± 5.6199	18.48934 ± 2.451	28.44945 ± 5.7305	28.27723 ± 7.6008	24.49685 ± 7.7459
Butyric acid	54.51725 ± 16.677	50.97640 ± 10.485	43.38789 ± 9.5930	39.37725 ± 1.0658	40.96370 ± 12.8063
Isovaleric acid	28.35359 ± 11.478	25.92176 ± 6.9173	24.11544 ± 6.0728	25.88972 ± 8.1664	21.20475 ± 6.2528
2-Methylbutyric acid	0.91781 ± 0.5654	0.92756 ± 0.2038	1.04895 ± 0.2700	1.07272 ± 0.2822	1.32042 ± 0.3838

**Table 4:** Relative percentages of volatile compounds of interest in the headspace of the chicken noodle soup sample packaged in metal cans.

Compounds	Soup					Coating			
	Day 0	Day 20	Day 25	Day 30	Day 35	Day 20	Day 25	Day 30	Day 35
Sulfur									
Hydrogen sulfide	0.63964 ± 0.301	0.03332 ± 0.013	0.04461 ± 0.016	0.04881 ± 0.025	0.12810 ± 0.026	0.00000	0.00000	0.00000	0.00000
Dimethyl sulfide	95.92298 ± 7.9326	97.74751 ± 5.087	98.37237 ± 10.951	98.12923 ± 13.440	96.53006 ± 9.734	62.50000 ± 8.945	56.80120 ± 0.766	64.42742+6.777	72.99125 ± 1.173
Methyl mercaptan	1.38542 ± 0.386	0.00208 ± 0.0	0.01371 ± 0.009	0.01836 ± 0.007	0.06934 ± 0.043	4.56647 ± 1.604	9.28625 ± 0.785	5.55883 ± 2.082	3.83850 ± 1.114
Propyl mercaptan	0.41307 ± 0.123	0.31977 ± 0.0	0.12318 ± 0.064	0.18966 ± 0.041	0.45311 ± 0.079	3.22254 ± 0.014	7.10015 ± 0.019	4.61599 ± 0.020	4.67383 ± 0.020
Methional	1.34315 ± 0.445	1.35907 ± 0.0	1.05745 ± 0.095	1.33713 ± 0.271	2.39046 ± 0.740	5.99711 ± 1.040	6.03513 ± 1.485	4.61599	6.14558
Dimethyl trisulfide	0.27866 ± 0.092	0.52424 ± 0.054	0.37824 ± 0.105	0.26705 ± 0.053	0.40635 ± 0.114	23.59827 ± 2.298	20.77728 ± 5.624	20.78177 ± 1.827	12.29117 ± 3.262
2-Isobutylthiazole	0.01708 ± 0.011	0.01401 ± 0.005	0.01045 ± 0.003	0.00976 ± 0.053	0.02258 ± 0.009	0.11561 ± 0.072	0.00000	0.00000	0.05967 ± 0.040
Acid									
Acetic acid	2.5088 ± 0.676	7.7631 ± 0.342	4.5618 ± 1.005	11.5821 ± 4.013	5.8529 ± 0.531	36.6343 ± 16.068	35.5883+14.484	19.6629 ± 3.820	13.9519 ± 2.069
Propionic acid	13.7026 ± 5.620	47.9120 ± 3.378	44.9045 ± 4.831	50.0424 ± 9.343	51.8469 ± 8.470	27.8294 ± 13.746	26.5973+5.435	49.4382 ± 12.416	55.2069 ± 16.756
Butyric acid	54.5173 ± 16.678	17.9939 ± 8.506	32.3091 ± 5.486	24.2675 ± 5.876	24.9993 ± 0.904	10.6630 ± 5.659	11.3617+5.840	5.5056 ± 3.483	6.5421 ± 0.601
Isovaleric acid	28.3536 ± 11.478	23.2917 ± 7.657	14.3447 ± 3.503	11.7923 ± 2.348	15.5468 ± 5.062	14.0836 ± 3.354	13.0963+3.354	15.9551 ± 1.112	11.2150 ± 4.606
2-Methylbutyric acid	0.9178 ± 0.118	3.0393 ± 0.801	3.8800 ± 0.982	2.3156 ± 0.537	1.7541 ± 0.432	10.7897 ± 4.857	13.3565+4.857	9.4382 ± 5.899	13.0841 ± 8.278

**Table 5:** Relative percentages of volatile compounds of interest in the headspace of the chicken breast group.

Compounds	Soup					Coating			
	Day 0	Day 5	Day 8	Day 11	Day 14	Day 5	Day 8	Day 11	Day 14
Sulfur									
Hydrogen sulfide	0.4578 ± 0.176	0.1944 ± 0.041	0.0325 ± 0.027	0.0010 ± 0.001	0.0000	2.9627 ± 0.293	0.1141 ± 0.076	0.0000	0.0000
Dimethyl sulfide	97.6984 ± 8.781	96.8832 ± 4.374	97.9831 ± 3.589	97.3590 ± 1.377	97.8116 ± 7.352	61.5234 ± 5.153	54.1358 ± .209	70.0915 ± 5.205	68.4108 ± 5.504
Methyl mercaptan	0.3704 ± 0.077	0.1729 ± 0.026	0.0618 ± 0.037	0.0083 ± 0.005	0.0000	0.0000	0.0000	0.0000	0.0000
Propyl mercaptan	0.1828 ± 0.032	0.4296 ± 0.034	0.2946 ± 0.027	0.4671 ± 0.013	0.1970 ± 0.005	4.0481 ± 1.418	6.4461 ± 0.608	4.9848 ± 0.369	8.0221 ± 1.433
Methional	0.9790 ± 0.198	0.9614 ± 0.046	0.8907 ± 0.057	1.1151 ± 0.267	1.2447 ± 0.097	3.3539 ± 0.372	3.5938 ± 0.941	3.5241 ± 0.326	5.6014 ± 1.661
Dimethyl trisulfide	0.2863 ± 0.051	1.3448 ± 0.022	0.7267 ± 0.314	1.0387 ± 0.096	0.7320 ± 0.098	27.6034 ± 2.151	35.6532 ± 1.445	21.1373 ± 1.787	17.7812 ± 1.802
2-Isobutylthiazole	0.0252 ± 0.005	0.0137 ± 0.003	0.0105 ± 0.002	0.0107 ± 0.004	0.0147 ± 0.003	0.5085 ± 0.225	0.0570 ± 0.038	0.2624 ± 0.085	0.1845 ± 0.043
Acid									
Acetic acid	0.9205 ± 0.532	11.8158 ± 1.803	4.9744 ± 1.644	18.5133 ± 3.093	5.2180 ± 1.868	5.8050 ± 0.960	5.9826 ± 2.795	2.7096 ± 1.169	9.0439 ± 0.851
Propionic acid	8.8477 ± 1.627	21.1616 ± 1.077	23.0069 ± 1.211	23.5409 ± 3.330	32.8562 ± 17.433	61.4503 ± 13.376	65.5531 ± 4.909	69.9746 ± 0.576	67.7290 ± 17.854
Butyric acid	72.4658 ± 22.474	47.5406 ± 7.938	53.9427 ± 8.053	42.3999 ± 2.988	46.8022 ± 6.116	12.4930 ± 5.504	2.9998 ± 1.977	3.9119 ± 0.779	4.5553 ± 0.984
Isovaleric acid	17.2242 ± 5.053	13.7682 ± 0.940	14.0154 ± 1.797	12.1915 ± 1.025	12.6990 ± 1.762	10.7646 ± 1.494	13.3458 ± 2.045	12.3624 ± 0.694	10.3955 ± 1.518
2-Methylbutyric acid	0.5419 ± 0.102	5.7137 ± 0.508	4.0606 ± 0.209	3.3545 ± 0.444	2.4246 ± 0.627	9.4871 ± 1.371	12.1186 ± 1.040	11.0415 ± 0.830	8.2763 ± 1.201

**Table 6:** Relative percentages of volatile compounds of interest in the headspace of the carrot group.

Compounds	Soup					Coating			
	Day 0	Day 5	Day 8	Day 11	Day 14	Day 5	Day 8	Day 11	Day 14
Sulfur									
Hydrogen sulfide	1.0558 ± 0.522	0.0361 ± 0.007	0.0260 ± 0.006	0.0099 ± 0.007	0.0079 ± 0.001	0.0000 ± 0.0	0.0399 ± 0.015	0.0551 ± 0.312	0.0787 ± 0.04
Dimethyl sulfide	97.2283 ± 6.707	98.3409 ± 3.049	98.5195 ± 13.364	98.0499 ± 0.469	98.3555 ± 7.463	20.6083 ± 3.081	40.1676 ± 1.596	58.0725 ± 1.735	63.366 ± 4.09
Methyl mercaptan	0.4241 ± 0.078	0.0882 ± 0.018	0.0675 ± 0.021	0.0181 ± 0.003	0.0046 ± 0.0	0.0000 ± 0.0	0.0000 ± 0.0	0.0000 ± 0.0	0.0000 ± 0.0
Propyl mercaptan	0.1899 ± 0.046	0.2644 ± 0.008	0.2483 ± 0.035	0.3076 ± 0.005	0.2273 ± 0.127	3.5468 ± 1.672	4.8028 ± 0.294	10.1147 ± 1.845	4.2821 ± 1.739
Methional	0.8040 ± 0.146	0.7245 ± 0.004	0.8322 ± 0.093	0.9136 ± 0.005	0.7831 ± 0.215	4.2796 ± 0.537	2.8378 ± 0.544	5.2501 ± 0.367	4.8326 ± 0.891
Dimethyl trisulfide	0.2772 ± 0.067	0.5310 ± 0.032	0.2958 ± 0.031	0.6821 ± 0.002	0.6083 ± 0.144	71.4763 ± 13.575	51.9076 ± 1.037	26.2965 ± 13.76	26.4529 ± 13.939
2-Isobutylthiazole	0.0207 ± 0.007	0.0149 ± 0.004	0.0108 ± 0.006	0.0187 ± 0.002	0.0133 ± 0.002	0.0889 ± 0.075	0.2444 ± 0.130	0.2111 ± 0.147	0.9875 ± 0.245
Acid									
Acetic acid	0.8717 ± 0.340	3.4231 ± 0.193	4.1980 ± 0.488	5.4704 ± 1.842	6.4722 ± 0.545	20.0352 ± 9.950	3.2241 ± 3.728	11.9418 ± 8.058	8.4337 ± 1.842
Propionic acid	9.7466 ± 1.982	21.9240 ± 0.913	24.5297 ± 2.525	24.5891 ± 0.312	27.9681 ± 17.433	33.6443 ± 14.449	63.0888 ± 4.723	58.5455 ± 11.71	62.4785 ± 10.740
Butyric acid	73.3300 ± 16.563	54.6923 ± 6.345	53.8307 ± 20.260	50.7723 ± 0.327	50.0304 ± 11.380	5.6074 ± 1.627	4.6305 ± 1.592	5.4982 ± 4.945	7.5904 ± 10.826
Isovaleric acid	15.2559 ± 3.980	13.5936 ± 1.678	12.8011 ± 3.655.	13.1887 ± 0.118	12.8371 ± 3.807	21.8698 ± 3.514	13.9445 ± 1.393	14.9527 ± 3.302	12.8399 ± 7.969
2-Methylbutyric acid	0.7958 ± 0.211	6.3670 ± 0.716	4.6405 ± 0.763	5.9794 ± 0.133	2.6922 ± 0.451	18.8433 ± 3.804	15.1121 ± 1.048	9.0618 ± 5.469	8.6575 ± 3.563

**Table 7:** Relative percentages of volatile compounds of interest in the headspace of the celery group.

Compounds	Soup						Coating				
	Day 0	Day 5	Day 10	Day 15	Day 20	Day 25	Day 5	Day 10	Day 15	Day 20	Day 25
Sulfur											
Hydrogen sulfide	1.3144 ± 0.568	0.0204 ± 0.002	0.0635 ± 0.024	0.0006 ± 0.0	0.0100 ± 0.0	0.0070 ± 0.0	0.0000	0.3830 ± 0.315	0.0000	0.0000	0.0000
Dimethyl sulfide	97.0856 ± 7.309	98.2439 ± 15.953	97.7223 ± 10.621	98.0306 ± 7.196	98.8623 ± 3.487	98.5578 ± 5.042	83.7037 ± 33.889	82.5855 ± 8.988	71.4468 ± 12.144	68.6761 ± 10.122	78.4995 ± 9.193
Methyl mercaptan	0.4356 ± 0.071	0.0359 ± 0.011	0.0000	0.0032 ± 0.001	0.0000 ±	0.0204 ± 0.0	1.8519 ± 0.0	0.0000	0.0000	0.4624 ± 0.103	0.0000
Propyl mercaptan	0.1601 ± 0.039	0.2394 ± 0.051	0.3467 ± 0.155	0.1656 ± 0.016	0.1451 ± 0.065	0.1986 ± 0.039	14.4444 ± 8.988	8.0301 ± 1.163	5.7390 ± 1.502	1.8325 ± 0.908	2.4501 ± 1.235
Methional	0.7220 ± 0.172	0.7479 ± 0.158	1.0717 ± 0.156	0.7569 ± 0.037	0.7205 ± 0.214	0.9405 ± 0.056	0.0000	8.1395 ± 2.285	4.0102 ± 1.403	4.5042 ± 1.987	4.9953 ± 0.057
Dimethyl trisulfide	0.2646 ± 0.056	0.6974 ± 0.149	0.7812 ± 0.192	1.0358 ± 0.095	0.2496 ± 0.045	0.2599 ± 0.056	0.0000	0.8208 ± 0.214	18.6056 ± 4.846	24.4562 ± 2.723	13.9221 ± 3.229
2-Isobutylthiazole	0.0176 ± 0.008	0.0150 ± 0.011	0.0146 ± 0.019	0.0073 ± 0.003	0.0125 ± 0.007	0.0157 ± 0.005	0.0000	0.0410 ± 0.096	0.1984 ± 0.283	0.0685 ± 0.069	0.1330 ± 0.399
Acid											
Acetic acid	0.9216 ± 0.774	28.3006 ± 7.349	58.0604 ± 15.993	1.7914 ± 0.696	1.9078 ± 0.481	2.4792 ± 1.329	0.0000	18.5654 ± 6.835	3.2787 ± 3.449	17.4753 ± 5.464	10.1863 ± 9.394
Propionic acid	9.1480 ± 1.726	23.1800 ± 4.720	16.1048 ± 1.821	29.3611 ± 1.290	27.6014 ± 17.433	27.0413 ± 2.775	0.0000	59.1842 ± 13.474	63.4873 ± 14.584	49.0344 ± 13.472	42.3702 ± 10.384
Butyric acid	74.0356 ± 16.821	26.7427 ± 5.619	14.2355 ± 3.179	54.4290 ± 10.230	56.7168 ± 7.356	55.0040 ± 14.184	9.0909 ± 1.993	5.8087 ± 3.277	10.0064 ± 2.895	10.9750 ± 1.554	19.2628 ± 1.744
Isovaleric acid	15.1312 ± 3.930	10.5791 ± 2.595	5.2405 ± 2.588	11.6262 ± 1.409	12.5244 ± 2.548	14.3687 ± 3.881	0.0000	9.8031 ± 3.797	12.5186 ± 6.110	8.5728 ± 2.920	13.2382 ± 1.942
2-Methylbutyric acid	0.7635 ± 0.177	11.1977 ± 1.420	6.3588 ± 2.109	2.7924 ± 0.115	1.2495 ± 0.413	1.1069 ± 0.283	90.9091 ± 4.981	6.6385 ± 2.166	10.7090 ± 1.341	13.9425 ± 4.333	14.9425 ± 1.625

**Table 8:** Relative percentages of volatile compounds of interest in the headspace of the broth and salt group.

Compounds	Soup						Coating				
	Day 0	Day 5	Day10	Day 15	Day 20	Day 25	Day 5	Day10	Day 15	Day 20	Day 25
Sulfur											
Hydrogen sulfide	1.9890 ± 0.124	0.0000	0.0000	0.0104 ± 0.0	0.0006 ± 0.010	0.0000	0.0000	0.0000	0.0000	0.0000	0.0000
Dimethyl sulfide	96.5140 ± 5.810	97.3474 ± 18.821	96.0858 ± 7.696	97.8008 ± 5.588	98.5836 ± 5.734	98.8025 ± 7.857	52.5563 ± 12.783	68.0658 ± 7.624	68.3667 ± 11.555	67.1366 ± 7.411	77.4651 ± 5.091
Methyl mercaptan	0.4177 ± 0.286	0.0732 ± 0210	0.1574 ± 0.0	0.0264 ± 0.001	0.0007 ± 0.0	0.0147 ± 0.0	5.7174 ± 00	0.0000	0.0000	0.0450 ± 0.154	0.0000
Propyl mercaptan	0.1615 ± 0.050	0.5539 ± 0.114	0.9544 ± 0.091	0.2298 ± 0.025	0.2062 ± 0.068	0.1614 ± 0.040	22.0451 ± 7.456	14.9950 ± 1.560	4.3656 ± 4.344	2.7899 ± 0.069	3.5919 ± 0.677
Methional	0.5915 ± 0.224	1.0045 ± 0.323	1.5345 ± 1.253	0.7660 ± 0.014	0.8156 ± 0.156	0.7624 ± 0.213	8.4112 ± 0.0	9.1372 ± 2.854	4.7600 ± 1.742	4.5298 ± 0.850	7.1608 ± 1.727
Dimethyl trisulfide	0.3055 ± 0.074	0.9821 ± 0451	1.2310 ± 0.086	1.1521 ± 0.012	0.3788 ± 0.075	0.2451 ± 0.048	11.2699 ± 0.0	7.6101 ± 0.412	22.2086 ± 5.947	25.2887 ± 7.862	11.7822 ± 6.268
2-Isobutylthiazole	0.0208 ± 0.006	0.0388 ± 0.010	0.0369 ± 0.008	0.0147 ± 0.002	0.0145 ± 0.004	0.0138 ± 0.009	0.0000	0.1919 ± 0.030	0.2992 ± 0.289	0.2100 ± 0.093	0.0000
Acid						±0					
Acetic acid	1.2315 ± 0.814	57.1060 ± 9.296	74.9428 ± 5.891	2.4128 ± 0.209	4.0605 ± 1.495	2.3587 ± 0.773	68.0877 ± 0.0	57.8476 ± 0.001	29.8562 ± 0.448	18.4020 ± 9.646	17.8815 ± 3.229
Propionic acid	9.3026 ± 2.373	13.5228 ± 15.338	6.5651 ± 5.682	21.4191 ± 2.072	28.8108 ± 17.433	24.2570 ± 5.755	8.6989 ± 0.0	20.3127 ± 11.696	45.4029 ± 11.898	44.7514 ± 22.492	51.1274 ± 10.310
Butyric acid	72.7985 ± 15.798	13.8601 ± 7.832	12.3582 ± 0.276	58.9311 ± 10.433	52.0710 ± 13.676	57.9207 ± 15.479	14.2435 ± 6.291	12.2068 ± 0.457	5.3159 ± 10.950	8.6698 ± 2.690	5.7158 ± 1.047
Isovaleric acid	15.6270 ± 3.705	8.5811 ± 6.132	2.8167 ± 2.259	14.2695 ± 2.459	13.5130 ± 2.999	14.5155 ± 4.530	5.1996 ± 0.0	5.4268 ± 3.023	10.9829 ± 2.725	12.4097 ± 5.813	11.5364 ± 1.403
2-Methylbutyric acid	1.0404 ± 0.258	6.9300 ± 3.369	3.3172 ± 4.083	2.9676 ± 0.408	1.5446 ± 0.291	0.9481 ± 0.222	3.7703 ± 0.1.961	4.2061 ± 2.417	8.4420 ± 2.269	15.7671 ± 1.992	13.7389 ± 4.843

**Table 9:** Relative percentages of volatile compounds of interest in the headspace of soup samples packaged with distilled water.

Compounds	Chicken breast	Frozen carrot	Fresh celery	Egg noodles
Sulfur				
Hydrogen sulfide	1.8379 ± 0.071	0.0000	0.0000	0.0000
Dimethyl sulfide	96.6383 ± 4.067	98.3627 ± 2.245	99.4831 ± 4.313	98.8633 ± 2.854
Methyl mercaptan	1.0299 ± 0.080	0.0000	0.0000	0.0000
Propyl mercaptan	0.0767 ± 0.002	0.5649 ± 0.071	0.1352 ± 0.017	0.2197 ± 0.037
Methional	0.1900 ± 0.010	0.3472 ± 0.046	0.1323 ± 0.021	0.4127 ± 0.039
Dimethyl trisulfide	0.2206 ± 0.004	0.7091 ± 0.040	0.2448 ± 0.024	0.4848 ± 0.012
2-Isobutylthiazole	0.0066 ± 0.002	0.0160 ± 0.005	0.0046 ± 0.001	0.0195 ± 0.005
Acid				
Acetic acid	0.6710 ± 0.043	31.7164 ± 3.615	4.1493 ± 0.285	8.0447 ± 0.381
Propionic acid	2.4773 ± 0.041	28.5657 ± 2.706	47.9221 ± 2.558	26.2295 ± 0.687
Butyric acid	89.3723 ± 8.549	22.1382 ± 2.655	27.5668 ± 4.487	27.3626 ± 0.659
Isovaleric acid	7.0134 ± 0.536	14.2011 ± 0.531	16.2126 ± 0.225	35.3563 ± 1.825
2-Methylbutyric acid	0.4660 ± 0.042	3.3787 ± 0.566	4.1493 ± 0.285	3.0068 ± 0.102

**Table 10:** Relative percentages of volatile compounds of interest in the headspace of the cysteine group.

Compounds	Soup					Coating			
	Day 0	Day 5	Day 8	Day 11	Day 14	Day 5	Day 8	Day 11	Day 14
Sulfur									
Hydrogen sulfide	18.7633 ± 3.966	26.1371 ± 2.120	13.8014 ± 0.385	11.9221 ± 1.799	13.4230 ± 1.565	0.0902 ± 0.0	0.0000	0.0000	0.0000
Dimethyl sulfide	78.7910 ± 4.921	69.1739 ± 4.541	83.0937 ± 2.474	84.6080 ± 4.094	83.3511 ± 11.527	38.3857 ± 2.980	43.6898 ± 1.896	45.0700 ± 2.823	61.6165 ± 5.259
Methyl mercaptan	1.5676 ± 0.086	3.3727 ± 0.258	2.0561 ± 0.0	1.9997 ± 0.263	1.5502 ± 0.0	0.0000	0.0000	0.0000	0.0000
Propyl mercaptan	0.1523 ± 0.027	0.2947 ± 0.002	0.2390 ± 0.040	0.3001 ± 0.035	0.2290 ± 0.070	5.1336 ± 1.591	8.7876 ± 1.659	12.9011 ± 1.558	6.5414 ± 1.909
Methional	0.4970 ± 0.127	0.5671 ± 0.026	0.5720 ± 0.016	0.7796 ± 0.034	0.7193 ± 0.240	2.6835 ± 0.817	5.1970 ± 0.207	7.4474 ± 1.248	4.5929 ± 2.068
Dimethyl trisulfide	0.2161 ± 0.049	0.4458 ± 0.002	0.2321 ± 0.038	0.3822 ± 0.029	0.7186 ± 0.461	53.4949 ± 6.756	41.8709 ± 3.443	34.3386 ± 3.82	26.5633 ± 1.978
2-Isobutylthiazole	0.0126 ± 0.004	0.0087 ± 0.003	0.0058 ± 0.002	0.0083 ± 0.002	0.0087 ± 0.021	0.2121 ± 0.191	0.4547 ± 0.201	0.2429 ± 0.243	0.6860 ± 0.457
Acid									
Acetic acid	1.8988 ± 0.444	5.7033 ± 0.016	5.1748 ± 0.255	3.7842 ± 0.433	3.8064 ± 1.691	6.1574 ± 3.863	32.4146 ± 14.962	35.8380 ± 26.113	8.0670 ± 5.243
Propionic acid	7.1226 ± 1.391	27.7457 ± 2.261	20.0361 ± 1.767	29.3895 ± 1.610	30.1402 ± 17.433	55.7725 ± 6.072	38.5471 ± 4.508	41.5713 ± 7.905	58.3968 ± 12.261
Butyric acid	72.7024 ± 12.059	26.3920 ± 4.217	47.1877 ± 19.270	35.8582 ± 3.090	38.3897 ± 3.345	8.1956 ± 0.741	4.0752 ± 1.664	3.0134 ± 2.240	8.1515 ± 2.486
Isovaleric acid	17.3739 ± 3.627	16.8975 ± 2.276	15.3075 ± 2.311	15.8883 ± 3.535	15.3507 ± 4.572	16.6904 ± 4.076	15.8874 ± 3.081	13.1885 ± 12.083	15.1869 ± 0.592
2-Methylbutyric acid	0.9023 ± 0.275	23.2615 ± 3.254	12.2938 ± 1.830	15.0798 ± 2.032	12.3130 ± 4.666	13.1842 ± 0.855	9.0757 ± 0.315	6.3888 ± 2.182	10.1979 ± 7.543

**Table 11:** Relative intensities of volatile compounds of interest in the headspace of the cysteine buffer group.

Compounds	Soup					Coating			
	Day 0	Day 20	Day 25	Day 30	Day 35	Day 20	Day 25	Day 30	Day 35
Sulfur									
Hydrogen sulfide	8.6716 ± 0.823	10.6678 ± 1.715	0.0363 ± 0.008	35.3372 ± 1.511	50.3211 ± 4.241	0.0000	0.2397 ± 0.0	0.0000	0.0000
Dimethyl sulfide	90.8767 ± 11.372	88.3451 ± 3.829	99.5371 ± 5.175	64.2797 ± 5.169	49.3925 ± 4.602	24.8011 ± 2.857	33.6898 ± 0.111	67.3115 ± 6.705	79.5743 ± 7.518
Methyl mercaptan	0.0000	0.0000	0.0000	0.0761 ± 0.016	0.0551 ± 0.0	0.0000	0.7376 ± 0.0	0.0000	0.0000
Propyl mercaptan	0.1961 ± 0.0	0.1905 ± 0.004	0.0600 ± 0.015	0.1010 ± 0.001	0.0646 ± 0.012	13.7794 ± 4.358	24.4330 ± 4.223	9.5845 ± 0	5.2523 ± 1.922
Methional	0.1104 ± 0.0	0.3019 ± 0.089	0.1292 ± 0.014	0.1332 ± 0.014	0.1441 ± 0.013	6.1935 ± 2.134	15.2683 ± 1.235	10.6177 ± 9.145	8.0673 ± 1.064
Dimethyl trisulfide	0.1415 ± 0.035	0.4931 ± 0.048	0.2349 ± 0.019	0.0713 ± 0.014	0.0216 ± 0.007	54.6203 ± 19.873	25.2259 ± 5.071	12.1785 ± 0.945	7.0717 ± 4.016
2-Isobutylthiazole	0.0037 ± 0.004	0.0015 ± 0.008	0.0026 ± 0.001	0.0015 ± 0.006	0.0011 ± 0.007	0.6058 ± 0.190	0.4057 ± 0.092	0.3078 ± 0.835	0.0343 ± 0.206
Acid									
Acetic acid	11.4738 ± 7.275	1.0360 ± 1.556	11.4325 ± 0.588	10.3737 ± 4.858	20.0512 ± 10.026	43.5581 ± 5.563	67.1077 ± 2.537	65.1894 ± 5.144	50.2573 ± 17.953
Propionic acid	35.2311 ± 7.728	81.4059 ± 4.090	43.8179 ± 2.419	63.0928 ± 5.979	53.2401 ± 17.433	26.6597 ± 4.373	13.8994 ± 0.347	13.9729 ± 4.918	30.0172 ± 12.865
Butyric acid	41.2898 ± 11.694	13.0429 ± 0.175	40.7208 ± 15.314	21.0567 ± 4.162	10.8529 ± 1.989	6.3938 ± 0.805	2.8647 ± 0.154	8.4115 ± 7.125	4.1166 ± 6.861
Isovaleric acid	8.7870 ± 1.495	2.2947 ± 0.108	2.4107 ± 1.945	4.0979 ± 2.384	9.5529 ± 1.064	15.7002 ± 2.204	12.0175 ± 2.980	6.3087 ± 2.815	5.9463 ± 6.747
2-Methylbutyric acid	3.2183 ± 1.553	2.2205 ± 0.121	1.6180 ± 0.466	1.3789 ± 0.606	6.3029 ± 0.532	7.6883 ± 2.510	4.1107 ± 1.336	6.1175 ± 2.763	9.6627 ± 4.345

## Data Availability

Data supporting this manuscript can be found at The Ohio State University Library, Columbus, OH 43210, USA.
